# Clinical parameters predictors of malignant transformation of recurrent parotid pleomorphic adenoma

**DOI:** 10.1038/s41598-023-29714-6

**Published:** 2023-03-20

**Authors:** Yu Pei, Wenlu Li

**Affiliations:** Department of Stomatology, The First Affiliated Hospital of Zhengzhou University, Zhengzhou, China

**Keywords:** Oral cancer, Signs and symptoms, Oral diseases

## Abstract

Malignant transformation (MT) in recurrent parotid pleomorphic adenomas (PAs) is rare; therefore its occurrence lacks reliable predictive factors. Our goal was to clarify the predictors for MT of recurrent parotid PAs based on preoperative clinical parameters. Patients with a clinical diagnosis of recurrent parotid PA were retrospectively enrolled. The association between clinicopathologic variables and MT of PA was assessed using univariate and multivariate analyses. MT occurred in 11.8% of the 467 patients. In univariate analysis, three or more previous recurrences, newly developed facial nerve paralysis, difficulty in mouth opening, tumors with the largest tumor diameter ≥ 2.4 cm, and abnormal neck lymph node enlargement were associated with MT occurrence. Further, multivariate analysis showed that three or more previous recurrences, newly developed facial nerve paralysis, difficulty in mouth opening, and abnormal neck lymph node enlargement were independently related to MT. MT of recurrent PA was not uncommon. Clinical signs of malignancy included newly developed facial nerve paralysis, difficulty in mouth opening, three or more previous recurrences, and abnormal neck lymph node enlargement.

## Introduction

Pleomorphic adenoma (PA) is the most common benign tumor in salivary gland neoplasms^1^; surgical excision is the preferred treatment method. However, owing to pseudopodia extending through the capsule or tumor rupture during surgery, relapses occasionally occur^2^. Recurrence rates can be from 2 to 3% at 5 years and about 7% at 20 years after initial surgery^3^.

Malignant transformation (MT) occurs in 0–23% of recurrent tumors; the resulting cancer is also known as carcinoma ex pleomorphic adenoma (CXPA), which refers to the malignant component in recurrent PA^4^. There are some differences regarding treatments for CXPA and PA. Preoperative definitive diagnosis can assist in the development of an appropriate treatment plan; however, it is difficult to distinguish between CXPA and PA if there is no typical indication of malignancy^5^. Some clinicopathologic variables including age, tumor size, tumor location, and smoking have been found to have predictive value, but multivariate analyses on those studies have not been performed^6,7^.

Therefore, the current study aimed to clarify the predictors for MT of recurrent PAs based on preoperative clinical parameters.

## Patients and methods

### Ethical considerations

The institutional research committee (The First Affiliated Hospital of Zhengzhou University) approved this study, and all participants signed an informed consent agreement. All methods were performed in accordance with the relevant guidelines and regulations. All procedures performed in this study involving human participants were in accordance with the ethical standards of the institutional and/or national research committee and with the 1964 Helsinki Declaration and its later amendments or comparable ethical standards.

### Patient selection

From January 2000 to January 2022, consecutive medical records of adult patients with a clinical diagnosis of recurrent parotid gland PA were retrospectively reviewed in a tertiary teaching hospital (The First Affiliated Hospital of Zhengzhou University). Inclusion criteria were as follows: surgical excision was performed for the tumors at the same sites, and a previous PA diagnosis was confirmed pathologically by repeat review in our hospital. Patients with a history of other malignancies were excluded. Information regarding demography, medical history, symptoms, image analysis, pathology, and treatment was extracted and analyzed.

### Treatment principle

All patients underwent adequate imaging examination including ultrasound and computed tomography (CT). Fine needle aspiration was not routinely performed unless there were suspicious enlarged lymph nodes. All operations were implemented under general anesthesia and based on previous scars. Postoperative adjuvant treatment was suggested if there were adverse pathologic features such as lymph node metastasis or high-grade malignant components.

### Definition of important variables

All pathologic sections were re-reviewed by at least two head and neck pathologists. Abnormal lymph node enlargement on CT was defined as follows: an area with clear evidence of nonfat, low-density, or liquid components; largest diameter of node > 15 mm at level II and > 10 mm at other levels; and a ratio of the longest to smallest diameter ≤ 2 mm^8^.

### Statistical analysis

The association between clinicopathologic variables and MT of PA was assessed using univariate (chi-square test) and multivariate (logistic test) analyses. All statistical analyses were performed using SPSS 20.0, and a *p* < 0.05 was considered significant.

### Ethics approval and consent to participate

The First Affiliated Hospital of Zhengzhou University institutional research committee approved our study, and all participants signed an informed consent agreement for medical research before initial treatment. And all the related procedures were consistent with Ethics Committee regulations.

## Results

In total, 467 patients were enrolled for analysis (200 men, 267 women; median age 50 [range 20–76]) years. Smoking and drinking were noted in 68 and 35 patients, respectively. The number of previous recurrences was distributed as 0 in 203 patients, 1 in 113 patients, 2 in 78 patients, 3 in 45 patients, and 4 or more in 28 patients. The median duration between initial PA and current recurrence was 7 years with a range from 1 to 24 years. No patients received radiotherapy previously.

A local mass was noted in all patients. Facial nerve paralysis occurred in 143 patients; of those, 8 patients exhibited newly developed paralysis (unrelated to previous surgeries). Pain occurred in 24 patients, and difficulty in mouth opening developed in 16 patients.

Regarding imaging studies, solitary, no more than 3, and greater than 3 recurrent foci were confirmed via imaging in 128, 139, and 200 patients, respectively. The mean largest diameter of tumors was 2.4 ± 1.2 cm. Abnormal neck lymph node enlargement occurred in 59 patients.

Fifty-five patients had postoperative pathology confirmation of CXPA, and 10 patients had neck lymph node metastasis. Pathology types of the malignant components were distributed as adenoid cystic carcinoma (ACC) in 27 patients, salivary duct carcinoma (SDC) in 15 patients, high-grade mucoepidermoid carcinoma (MEC) in 8 patients, and nonspecific adenocarcinoma (NOSAC) in 5 patients. All patients underwent postoperative radiotherapy.

### Association between preoperative data and MT occurrence

Patients with 3 or more previous recurrences had a possibility of 34.2% of MT occurrence, which was significantly higher than those with 0–2 previous recurrences (*p* < 0.001). All patients with newly developed facial nerve paralysis had MT occurrence; a significantly higher rate than that in patients who did not experience paralysis (*p* < 0.001). Compared to 11.1% in patients without difficulty in mouth opening, 31.3% of the patients with difficulty in mouth opening had MT occurrence, which was a significant difference (*p* = 0.030). Patients with tumors with the largest tumor diameter of ≥ 2.4 cm had a rate of 17.7% of MT occurrence. This frequency was higher than in patients with tumors of the largest tumor diameter of < 2.4 cm (*p* < 0.001). MT occurred in 23.7% of the patients with abnormal neck lymph node enlargement, and in 10.0% of the patients without abnormal neck lymph node enlargement, which was a significant difference (*p* = 0.002). No other significant associations were noted (Table 1).

**Table 1:** Univariate analysis of the association between preoperative data and malignant transformation (MT) occurrence.

Variable	MT occurrence		*p*
	Yes (n = 55)	No (n = 412)	
Age			
< 50	20 (36.4%)	203 (49.3%)	
≥ 50	35 (63.6%)	209 (50.7%)	0.072
Sex			
Male	29 (52.7%)	171 (41.5%)	
Female	26 (47.3%)	241 (58.5%)	0.114
Smoker			
Yes	9 (16.4%)	59 (14.3%)	
No	46 (83.6%)	353 (85.7%)	0.687
Drinker			
Yes	5 (9.1%)	32 (7.7%)	
No	50 (90.9%)	380 (92.3%)	0.789
Number of previous recurrence			
0	10 (18.2%)	193 (46.8%)	
1–2	20 (36.4%)	171 (41.5%)	
3 or more	25 (45.4%)	48 (11.7%)	< 0.001
Duration between initial tumor and current recurrence (years)			
< 7	16 (29.1%)	214 (51.9%)	
≥ 7	39 (70.9%)	198 (48.1%)	0.001
Newly developed facial nerve paralysis			
Yes	8 (14.5%)	0	
No	47 (85.5%)	412 (100%)	< 0.001
Pain			
Yes	3 (5.5%)	21 (5.1%)	
No	53 (94.5%)	391 (94.9%)	1.000
Difficulty in mouth opening			
Yes	5 (9.1%)	11 (2.7%)	
No	50 (90.9%)	401 (97.3%)	0.030
Recurrence pattern			
Solitary	17 (30.9%)	111 (26.9%)	
2–3	17 (30.9%)	122 (29.6%)	
> 3	21 (38.2%)	179 (43.5%)	0.733
The largest tumor diameter (cm)			
< 2.4	18 (32.7%)	240 (58.3%)	
≥ 2.4	37 (67.3%)	172 (41.7%)	< 0.001
Abnormal neck lymph node enlargement			
Yes	14 (25.5%)	45 (10.9%)	
No	41 (74.5%)	367 (89.1%)	0.002

In further multivariate analysis, 3 or more previous recurrences (*p* = 0.012, 3.467 [1.227–9.021]), newly developed facial nerve paralysis (*p* = 0.009, 5.329 [1.836–19.226]), difficulty in mouth opening (*p* = 0.011, 3.295 [1.645–10.445]), and abnormal neck lymph node enlargement (*p* = 0.006, 4.476 [1.934–10.583]) were independently related to the occurrence of MT. Other variables were no longer significant (Table 2).

**Table 2:** Multivariate analysis of the association between preoperative data and malignant transformation occurrence.

Variable	OR[95%CI]	*p*
Number of previous recurrence		
0		
1–2	2.343 [0.774–8.227]	0.323
3 or more	3.467 [1.227–9.021]	0.012
Duration between initial tumor and current recurrence		
< 7 years		
≥ 7 years	4.275 [0.588–13.264]	0.437
Newly developed facial nerve paralysis		
No		
Yes	5.329 [1.836–19.226]	0.009
Difficulty in mouth opening		
No		
Yes	3.295 [1.645–10.445]	0.011
The largest tumor diameter		
< 2.4 cm		
≥ 2.4 cm	3.888 [0.743–13.284]	0.236
Abnormal neck lymph node enlargement		
No		
Yes	4.476 [1.934–10.583]	0.006

### Survival of CXPA

After follow-up with a median time of 56 (range 9–135) months, 35 patients had disease recurrence; local recurrence occurred in 21 patients, regional recurrence occurred in 14 patients, and distant metastasis occurred in 7 patients, of whom 6 had lung metastasis and 1 had both lung and liver metastasis. Twenty-seven patients died of the disease. The 5-year disease-specific survival and recurrence-free survival rates were 45% and 36%, respectively (Supplementary Table).

## Discussion

The most important finding in the current study was that MT of recurrent PA was not uncommon. Additionally, three or more previous recurrences, newly developed facial nerve paralysis, difficulty in mouth opening, and abnormal neck lymph node enlargement significantly increased the risk of MT.

CXPA is relatively uncommon^9^ and usually arises from long-lasting primary PA, which has a reported MT or recurrent PA risk of up to 24%^10^. In current study, all the cases were pathologically confirmed based on a previous diagnosis of PA in the same region. The three most common histologic types tended to be SDC, MEC, and ACC^11^, it was also supported by our finding. Considering the different treatment principles, it is important to distinguish CXPA from PA to improve preoperative planning for surgery.

Facial nerve paralysis commonly occurred after parotidectomy but was uncommon before parotidectomy. A review by Reinheimer et al.^12^ revealed that 1.6% of patients with benign salivary tumors presented with facial nerve paralysis; in malignant cases, 5.7% demonstrated facial nerve paralysis, a significant difference. Inaka et al.^13^ reported that malignant salivary tumors had a higher incidence of symptoms and signs than benign tumors. Symptoms were more frequent in higher grades of malignancies, and facial nerve paralysis was more commonly observed in ACC, which was inclined to adhere to surrounding tissues. Similar results were also described by Comoglu et al.^14^, who found that all patients with preoperative facial nerve paralysis were diagnosed with malignant tumors. These findings suggest that facial nerve paralysis is indicative of malignancy. However, facial nerve paralysis can also be found in cases of benign salivary gland tumors owing to the decompression. In the current study, nearly one-third of the patients presented with facial nerve paralysis before the operation, but most could be explained by having had previous operations. Newly developed facial nerve paralysis may indicate aggressive growth or change in the nature of the tumor. Our findings confirmed this speculation; MT was found in all patients with newly developed facial nerve paralysis. Moreover, the current study may be the first to present the significance of this symptom in predicting the occurrence of CXPA. A possible explanation may be that approximately half of the malignant parts of CXPA were ACC, while others were all high-grade malignancies which tended to invade surrounding neurovascular tissue.

Difficulty of mouth opening is usually the reflection of infectious diseases or involvement of masticatory or other muscles, and is likely to occur following radiotherapy for head and neck cancers^15,16^. However, this is rarely reported in recurrent PA. The current study revealed that difficulty of mouth opening was uncommon, only occurring in 3.4% of the cases. Notably, this symptom was associated with a 31.3% risk of MT. This finding shows the importance of preoperative communication between the doctor and the patient. Generally, malignant tumors can cause limited mouth opening, but physicians must be aware that previous surgical scars can also lead to difficulty of mouth opening.

The number of previous recurrences is another important variable. Yin et al.^6^ recently reported a total of 13 CXPAs found in 106 recurrent PAs, and the rates of MT in patients with 0, 1, 2, and 3 previous recurrences were 12.7%, 14.7%, 0%, and 0%, respectively (the difference was not significant). No other similar literature was available for reviewing. However, the current study revealed that more than three previous recurrences were independently associated with increased risk of MT. The difference might be explained by different sample sizes and inclusion criteria. The underlying mechanism might be related to molecular signals^17–19^.

Abnormal neck lymph node enlargement requires clinical attention. Preoperative puncture biopsy or intraoperative frozen section is strongly suggested. Although false positivity is common (rate of 68.9%), it should not be ignored, and our findings showed that lymph node enlargement was significantly related to MT, which was also confirmed by Seok et al.^7^. The value of CT in detecting lymph node metastasis in head and neck squamous cell carcinoma has been widely evaluated^20^. The classic feature is cystic changes within the lymph node; however, there might be different manifestations from lymph node metastasis in salivary malignancy. Moreover, ACC had a low lymph node metastasis rate, and it accounted for nearly 50% of the CXPAs. This could partially explain the high false positivity rate.

Other predictors have also been described including age > 50 years, significant smoking history, tumors larger than 2 cm, and minor salivary gland tumors^6,7^. Egal et al.^21^ reported there were significant correlations between the degree of malignancy and age (r = 0.242; *p* < 0.001), larger tumor diameter (r = 0.216; *p* < 0.001), and the estimated tumor volume (r = 0.214; *p* < 0.001). The tendency to occur in older age might be explained by the multihit theory of oncogenesis. Older patients also have an increased risk of somatic mutations. Carcinogenesis from smoking is well known, which was heavily implicated in many smoking-related malignancies. The findings from our study provide insight into the MT process, but also revealed that the MT process is complex. More basic research is needed to clarify this information.

Optimal management of recurrent PA remains unknown. Complete resection was recommended by the French Society of Otorhinolaryngology-Head and Neck Surgery^22^, but increased risk including facial nerve paralysis was associated with surgical treatment^23^. However, MT could occur in long-lasting recurrent PA. Balancing benefit and risk should be considered for formulating treatment plans varying from observation to radical excision^2^. Treatment planning of recurrent PA should carefully take MT into consideration if there are existing risk factors, and aggressive excision would be required when a CXPA is confirmed.

Limitations in the current study must be acknowledged. First, the study was retrospective, leading to inherent bias. Second, potential molecular mechanisms were not analyzed, and further basic study was required.

In summary, this study showed that MT of recurrent parotid PA was not uncommon. Clinical signs of malignancy included newly developed facial nerve paralysis, difficulty in mouth opening, three or more previous recurrences, and abnormal neck lymph node enlargement.

## Supplementary Information

Supplementary Table 1.

## Data Availability

All data generated or analyzed during this study are included in this published article. And the primary data could be achieved from the corresponding author.
