# Supplementary material for: Clinical parameters predictors of malignant transformation of recurrent parotid pleomorphic adenoma
Source: Sci Rep. 2023 Mar 20;13:4543. doi: 10.1038/s41598-023-29714-6 (PMC10027859; doi:10.1038/s41598-023-29714-6)
Supplement: Supplementary file 1 — Supplementary Table 1. [file 41598_2023_29714_MOESM1_ESM.doc]

Supplementary Table. Detailed information for the 55 patients with carcinoma ex pleomorphic adenoma.

| Case | Age | Sex* | Number of previous recurrence | Duration between initial tumor and current recurrence (Years) | Histologic type of malignant component% | Outcome |
| --- | --- | --- | --- | --- | --- | --- |
| 1 | 36 | M | 4 | 6 | MEC | 9 months, died |
| 2 | 44 | M | 0 | 4 | NOSAC | 34 months, died |
| 3 | 50 | F | 3 | 5 | SDC | 26 months, died |
| 4 | 70 | F | 4 | 10 | MEC | 15 months, died |
| 5 | 28 | M | 1 | 4 | NOSAC | 19 months, died |
| 6 | 49 | F | 2 | 7 | SDC | 33 months, died |
| 7 | 65 | F | 3 | 9 | SDC | 45 months, died |
| 8 | 36 | M | 1 | 6 | SDC | 54 months, died |
| 9 | 68 | F | 4 | 10 | MEC | 38 months, died |
| 10 | 32 | F | 2 | 3 | SDC | 43 months, died |
| 11 | 54 | F | 2 | 6 | SDC | 59 months, died |
| 12 | 44 | M | 1 | 12 | ACC | 23 months, died |
| 13 | 62 | F | 5 | 7 | MEC | 14 months, died |
| 14 | 49 | M | 3 | 7 | ACC | 16 months, died |
| 15 | 52 | M | 4 | 8 | NOSAC | 18 months, died |
| 16 | 50 | F | 0 | 5 | SDC | 22 months, died |
| 17 | 50 | M | 3 | 24 | ACC | 24 months, died |
| 18 | 29 | M | 0 | 3 | ACC | 36 months, died |
| 19 | 36 | M | 1 | 10 | SDC | 37 months, died |
| 20 | 58 | F | 4 | 12 | ACC | 33 months, died |
| 21 | 63 | M | 0 | 3 | MEC | 44 months, died |
| 22 | 64 | M | 4 | 7 | SDC | 19 minths, died |
| 23 | 47 | M | 3 | 7 | ACC | 16 months, died |
| 24 | 55 | M | 3 | 9 | MEC | 19 months, died |
| 25 | 58 | F | 0 | 4 | ACC | 22 months, alive |
| 26 | 67 | F | 1 | 3 | ACC | 25 months, alive |
| 27 | 38 | F | 0 | 11 | ACC | 44 months, alive |
| 28 | 53 | M | 3 | 12 | SDC | 34 months, alive |
| 29 | 54 | F | 0 | 7 | ACC | 33 months, alive |
| 30 | 58 | M | 0 | 7 | ACC | 45 months, alive |
| 31 | 59 | F | 3 | 10 | SDC | 32 months, alive |
| 32 | 27 | F | 3 | 9 | ACC | 45 months, alive |
| 33 | 60 | M | 1 | 1 | ACC | 56 months, alive |
| 34 | 36 | M | 0 | 7 | SDC | 65 months, alive |
| 35 | 74 | F | 4 | 10 | ACC | 45 months, alive |
| 36 | 65 | F | 0 | 10 | MEC | 32 months, alive |
| 37 | 45 | M | 1 | 11 | ACC | 56 months, alive |
| 38 | 54 | F | 3 | 8 | ACC | 135 months, alive |
| 39 | 37 | M | 1 | 8 | NOSAC | 77 months, alive |
| 40 | 57 | M | 2 | 4 | ACC | 44 months, alive |
| 41 | 56 | F | 1 | 9 | ACC | 88 months, alive |
| 42 | 50 | M | 2 | 9 | SDC | 90 months, alive |
| 43 | 36 | F | 5 | 7 | ACC | 67 months, alive |
| 44 | 65 | F | 1 | 5 | SDC | 57 months, alive |
| 45 | 62 | M | 3 | 8 | ACC | 68 months, alive |
| 46 | 63 | M | 3 | 9 | ACC | 76 months, alive |
| 47 | 48 | M | 3 | 11 | MEC | 54 months, alive |
| 48 | 71 | F | 2 | 6 | ACC | 66 months, alive |
| 49 | 69 | F | 3 | 12 | ACC | 44 months, alive |
| 50 | 58 | M | 1 | 9 | ACC | 32 months, alive |
| 51 | 49 | M | 4 | 9 | NOSAC | 103 months, alive |
| 52 | 64 | F | 2 | 6 | ACC | 39 months, alive |
| 53 | 37 | M | 1 | 7 | ACC | 17 months, alive |
| 54 | 56 | M | 2 | 7 | SDC | 17 months, alive |
| 55 | 76 | F | 4 | 11 | ACC | 19 months, alive |

* M: male; F: female;

% ACC: adenoid cystic carcinoma; SDC: salivary duct carcinoma; MEC: mucoepidermoid carcinoma; NOSAC: nonspecific adenocarcinoma
